# Review of PP2A Tumor Biology and Antitumor Effects of PP2A Inhibitor LB100 in the Nervous System

**DOI:** 10.3390/cancers13123087

**Published:** 2021-06-21

**Authors:** Jean-Paul Bryant, Adam Levy, John Heiss, Yeshavanth Kumar Banasavadi-Siddegowda

**Affiliations:** 1 Surgical Neurology Branch, National Institute of Neurological Disorders and Stroke, National Institutes of Health, Bethesda, MD 20892, USA; jxb1400@med.miami.edu (J.-P.B.); heissj@ninds.nih.gov (J.H.); 2 Miller School of Medicine, University of Miami, Miami, FL 33136, USA; adam.levy@med.miami.edu

**Keywords:** PP2A, LB100, nervous system, tumor biology, brain tumor, preclinical, clinical trial

## Abstract

**Simple Summary:**

Central and peripheral nervous system tumors represent a heterogenous group of neoplasms which often demonstrate resistance to treatment. Given that these tumors are often refractory to conventional therapy, novel pharmaceutical regimens are needed for successfully treating this pathology. One such therapeutic is the serine/threonine phosphatase inhibitor, LB100. LB100 is a water-soluble competitive protein phosphtase inhibitor that has demonstrated antitumor effects in preclinical and clinical trials. In this review, we aim to summarize current evidence demonstrating the efficacy of LB100 as an inhibitor of nervous system tumors. Furthermore, we review the involvement of the well-studied phosphatase, protein phosphatase 2A, in oncogenic cell signaling pathways, neurophysiology, and neurodevelopment.

**Abstract:**

Protein phosphatase 2A (PP2A) is a ubiquitous serine/threonine phosphatase implicated in a wide variety of regulatory cellular functions. PP2A is abundant in the mammalian nervous system, and dysregulation of its cellular functions is associated with myriad neurodegenerative disorders. Additionally, PP2A has oncologic implications, recently garnering attention and emerging as a therapeutic target because of the antitumor effects of a potent PP2A inhibitor, LB100. LB100 abrogation of PP2A is believed to exert its inhibitory effects on tumor progression through cellular chemo- and radiosensitization to adjuvant agents. An updated and unifying review of PP2A biology and inhibition with LB100 as a therapeutic strategy for targeting cancers of the nervous system is needed, as other reviews have mainly covered broader applications of LB100. In this review, we discuss the role of PP2A in normal cells and tumor cells of the nervous system. Furthermore, we summarize current evidence regarding the therapeutic potential of LB100 for treating solid tumors of the nervous system.

## 1. Introduction

Protein phosphatase 2A (PP2A) is an evolutionarily conserved, ubiquitous serine/threonine phosphatase that is implicated in a wide variety of regulatory cellular functions, including cell survival, DNA-damage response, tau dephosphorylation, and apoptosis [1]. PP2A is abundant in the mammalian cerebrum and is comprised of three distinct subunits: PP2A-A (structural subunit), PP2A-B (regulatory subunit), and PP2A-C (catalytic subunit) [2]. A comprehensive list of known isoforms of PP2A subunits is displayed in Table 1 [3].

Given its abundance in the central nervous system (CNS), the dysregulation of PP2A pathways can contribute to the pathogenesis of many neurodegenerative diseases [4,5,6,7]. PP2A has also received attention for its oncologic properties, suppressing tumor growth by inhibiting numerous growth and survival pathways [8,9]. Intriguingly, PP2A inhibition with the small molecule inhibitor LB100 inhibits tumor growth, opposite to the predicted effect of blocking a tumor suppressor like PP2A.

LB100 abrogation of PP2A is believed to act as an adjuvant, inhibiting tumor progression by chemo- and radiosensitizing tumor cells [10]. While the implications of LB100 for many conditions have been described, an updated, detailed, and unifying review of therapeutic strategies focusing on cancers of the nervous system has not been published. Here, we discuss the role of PP2A in the context of normal nervous system processes and nervous system tumorigenesis. Furthermore, we summarize existing evidence for the therapeutic potential of LB100 in treating solid tumors of the nervous system.

## 2. Role of PP2A in Cellular Signaling Pathways

Cell cycle initiation and apoptosis are regulated by an array of intricate signaling pathways. The normal functioning of key regulatory elements within these pathways is essential for maintaining physiologic cellular behavior, with aberrant expression often leading to significant cellular pathology. We review here the most critical pathways with known associations with PP2A.

### 2.1. Mechanistic Target of Rapamycin (mTOR)

The mTOR signaling pathway has been extensively studied because of its involvement in many diverse cellular processes and its oncologic implications. The mTOR pathway accumulates the necessary elements for continued cell division and is stimulated by amino acids, the products of cellular metabolism, and growth factors [11]. This pathway promotes anabolic processes including lipid synthesis and ribosome biogenesis while inhibiting catabolic processes such as autophagy [11]. Ribosomal S6 kinase (S6K) and the eIF4E binding protein 1 (4E-BP1) are the most well-characterized substrates of mTOR [12]. At low levels of mTOR activity, 4E-BP1 remains hypophosphorylated, promotes binding with eIF4E, and blocks translation initiation. Conversely, high mTOR activity leads to ample phosphorylation of 4E-BP1, the subsequent release of eIF4E, and the initiation of cap-dependent translation [13].

Signaling molecules activated upstream and downstream of mTOR are implicated in oncogenesis [14]. Receptor tyrosine kinases (RTKs) activate phosphatidylinositol 3-kinase (PI3K) and initiate a signaling cascade leading to the mTORC1 and mTORC2 complexes [14]. Genetic alterations or amplifications are the usual causes of the constitutive activation of upstream protein molecules leading to aberrant signal transduction through mTOR complexes. *EGFR* gene mutations activating mTOR have been studied in colorectal cancers, glioblastomas (GBM), and non-small-cell lung cancer (among others) [12].

Downstream effectors of the mTOR signaling pathway play a critical role in oncogenesis by regulating cancer cell survival, growth, and biomolecule synthesis. Nakamura et al. discovered that S6K1 is a mediator of glial cell transformation and that its knockdown reduced intracranial tumor size [15]. 4EBP-1 is thought to be an important molecular marker of cancer cell malignancy, and its expression correlated with poorer prognosis in a variety of human malignancies [16].

**Figure 1:**
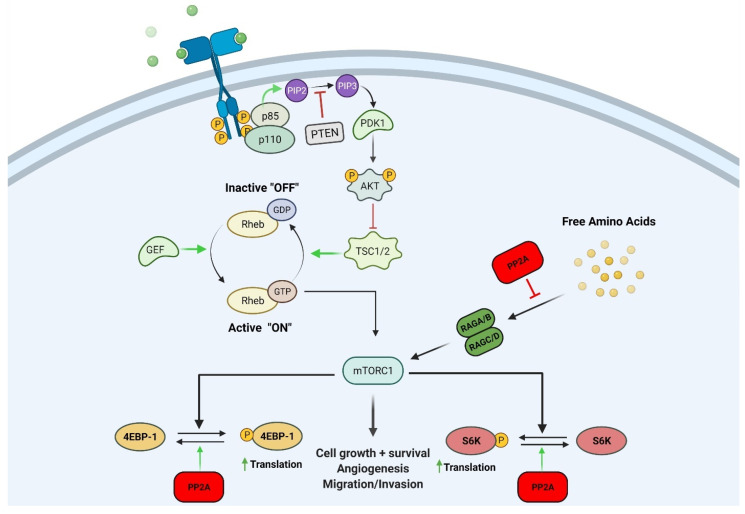
Schematic representation showing the mechanism in which PP2A can negatively regulate the mTOR pathway. PP2A can inhibit mTOR effectors 4EBP-1 and S6K by reversing their phosphorylation state. PP2A can also inhibit nutrient signaling to RAG complex proteins thereby inhibiting the mTOR pathway. Figure created with BioRender.com.

PP2A interacts with mTOR and other effectors in the mTOR signaling pathway (Figure 1) [14]. For example, Peterson et al. showed that FKBP12-rapamycin-associated protein inhibits the PP2A-mediated dephosphorylation of mTOR effectors 4EBP-1 and S6K (Figure 1) [17]. They also found that mTOR can inactivate PP2A and activate S6K. This activation induces ribosomal biogenesis and cell growth. PP2A was also reported to affect upstream members and regulatory elements of the PI3K/Akt/mTOR pathway [18]. TIPRL, an mTOR regulator, was shown to associate with the catalytic subunit of PP2A (PP2A-C) to stimulate mTOR, and this complex was required for TIPRL-dependent mTORC1 signaling [18].

The mTOR pathway has been implicated in nervous system tumorigenesis in inherited brain tumor predisposition syndromes and glioma [19]. Mutations in the gene of mTOR pathway protein PTEN predispose individuals to PTEN hereditary hamartoma tumor syndromes [20]. Those harboring these mutations are at greater risk of developing thyroid and breast cancer and, to a lesser extent, cerebellar dysplastic gangliocytomas, meningioma, and intracranial vascular malformations [20]. Glioma development is also associated with mTOR hyperactivation. *EGFR* gene amplification, often found in GBM, activates PI3K in nearly half of cases [21]. Furthermore, about 40% of patients with GBM have deleterious *PTEN* gene tumor mutations [22].

### 2.2. Wnt Signaling Pathway

Wnt signaling is a fundamental and essential pathway for embryonic development and normal postdevelopment homeostasis. Its primary function is to activate cell division promoters and regulate the proper initiation of the cell cycle [23]. Wnt signaling is activated by Wnt binding to the frizzled receptor, forming an intracellular complex that prevents degradation of the regulatory protein β-Catenin [24]. Free and intact β-Catenin can then bind to transcription factors that activate Wnt response genes (such as cyclin D1 and c-Myc) that promote cell division [25]. The absence of Wnt bound to its receptor allows β-Catenin to be degraded by a complex comprised of adenomatous polyposis coli (APC), axin, glycogen synthase 3β (GSK3β), and CK1 [23]. Wnt has been extensively studied in relation to its implications in human cancer because of its essential role in inducing embryonic cellular characteristics [26,27,28].

**Figure 2:**
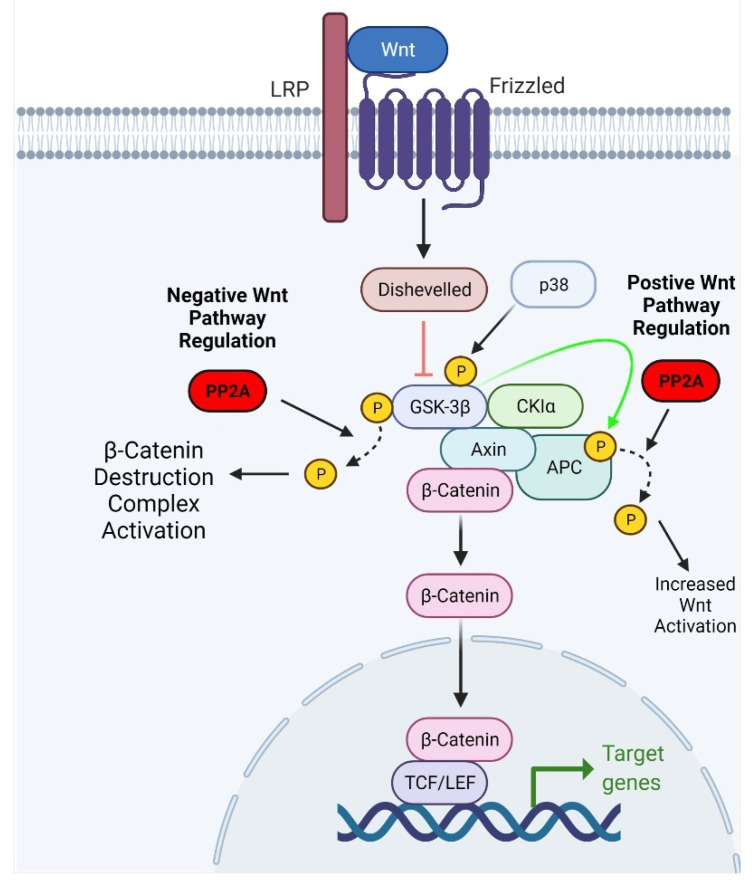
Schematic representation of the effect of PP2A on Wnt signaling. PP2A can reverse p38-dependent phosphorylation of GSK3β, thus activating its kinase activity. Activation of GSK3β leads to the ubiquitination and degradation of β-Catenin. Adapted from “Wnt Beta-Catenin Signaling Pathway,” by BioRender.com (2021). Retrieved from https://app.biorender.com/biorender-templates, accessed on 11 May 2021.

β-Catenin is the primary effector of the Wnt pathway, and its function is contingent on the phosphorylation state of its residues. Phosphorylation at the S37 and S33 residues facilitates the binding of β-transducin, a ubiquitin ligase, which targets the β-Catenin protein for degradation [29]. When phosphorylated, other members of the pathway, such as APC and axin, have increased affinity for β-Catenin and promote its degradation [30,31]. Phosphorylation of Wnt pathway proteins can be modified by protein phosphatases, including PP1 and PP2A [32,33]. PP2A can exert activating and inhibitory effects on the Wnt pathway. Positive regulation of Wnt signaling is largely attributable to a PP2A-B family regulatory subunit, PR55α [34,35]. PR55α has been shown to mediate PP2A-dependent dephosphorylation of β-Catenin [34]. In a study by Zhang et al., PR55α depletion resulted in β-Catenin degradation, suggesting that PR55α may positively regulate Wnt pathway signaling [34]. Interestingly, PP2A also demonstrated inhibitory effects on the Wnt signaling pathway. PP2A-mediated negative regulation of the Wnt signaling pathway occurs through proteins other than β-Catenin. For example, treatment with the PP2A inhibitor okadaic acid in mouse embryonic teratocarcinoma cells was reported to enhance Wnt signaling [36]. Furthermore, PP2A can dephosphorylate GSK3β and facilitate its kinase activity, resulting in phosphorylation and subsequent ubiquitination of β-Catenin (Figure 2) [37].

The upregulation of Wnt signaling has been implicated in GBM development because of its role in embryonic brain development and stem cell maintenance. Canonical Wnt signaling positively regulates transcription factor LEF1, which promotes cell migration and invasiveness in GBM and other cancers [38]. The Wnt pathway is critical to the maintenance of GBM stem cell properties. Furthermore, patients with GBM and aberrant signaling in the canonical Wnt pathway have a worse prognosis, and their tumors are more resistant to chemo- and radiotherapy [39,40]. Lastly, Wnt receptor Frizzled 4 is upregulated in invasive GBM cells and promotes the expression of epithelial to mesenchymal transition regulator SNAI1 [41].

### 2.3. Mitogen-Activated Protein Kinase (MAPK) Signaling Pathway

MAPKs mediate intracellular signaling and a wide variety of cellular processes, including differentiation, proliferation, and apoptosis [42,43]. This signaling pathway is comprised of four discrete cascades: the extracellular signal-related kinases (ERK1/2), Jun amino-terminal kinases (JNK1/2/3), p38-MAPK, and ERK5 [43]. This pathway is activated by upstream kinases (MAPKK kinases) which are stimulated by stress signals, cytokines, and growth signals [3]. The ERK1/2 pathway was the first cascade discovered and consequently is the best studied. It is a prominent promoter of cellular proliferation, survival, and transformation and therefore plays an important role in tumorigenesis [44]. Increased ERK expression occurs in many human malignancies, including breast cancer, colorectal cancer, and ovarian cancer (among others) [45].

**Figure 3:**
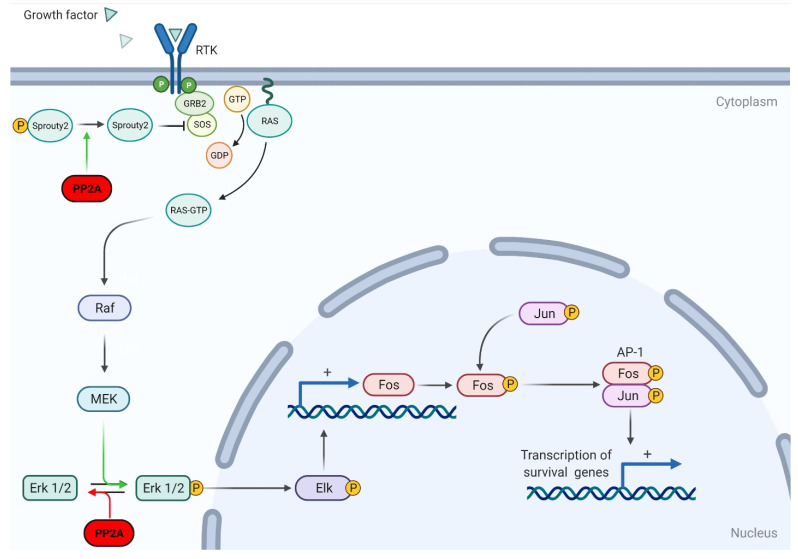
Schematic representation of PP2A regulation on the MAPK pathway. PP2A can dephosphorylate Sprouty2, thus inhibiting RTK-dependent activation of the MAPK/Erk pathway. PP2A can also dephosphorylate Erk1/2, preventing the activation of downstream effector Elk and preventing the transcription of transcription factors Fos and Jun. Adapted from “Ras Activation,” by BioRender.com (2021). Retrieved from https://app.biorender.com/biorender-templates, accessed on 11 May 2021.

While PP2A can positively regulate ERK/MAPK signaling, its main effect is to downregulate the ERK/MAPK pathway. Ugi et al. found that PP2A can bind to SHC, a Ras activator, and negatively regulate Ras stimulation [46]. Other studies have since supported the notion that PP2A downregulates the ERK/MAPK pathway through modifying various effectors. Lao et al. found that PP2A can dephosphorylate sprouty2, ultimately disrupting the activation of Ras and downregulating fibroblast growth factor (FGF)-mediated ERK/MAPK activation (Figure 3) [47].

Although the role of PP2A as a negative regulator of the ERK/MAPK pathway is well documented, there is also opposing evidence indicating that PP2A can positively regulate ERK signaling. Studies showed that the binding of PP2A regulatory subunit PR130 to SH2 domain-containing inositol 5-phosphatase 2 (known as SHIP2) is required for SHIP2-mediated stabilization of the EGFR [48]. Furthermore, Jaumot and Hancock demonstrated that PP1 and PP2A positively regulate the ERK/MAPK pathway by contributing to Raf-1 activation via dephosphorylation of its S259 residue [49]. In their in vitro experiments, the administration of PP2A inhibitors significantly inhibited Raf-1 activation. The opposing actions of PP2A on MAPK signaling are complex and further attention is needed to elucidate the net effect of PP2A on MAPK signaling.

MAPK pathway activation and deregulation are involved in brain tumor development and other nervous system pathologies. The product of the *NF1* gene, neurofibromin, acts as a GTPase-activating protein for MAPK pathway protein Ras [50]. Germline mutations in the *NF1* gene cause Neurofibromatosis type 1 (NF1). Mutation or deletion of the second copy of the NF1 gene within the cell results in the loss of neurofibromin, increased Ras activation, and tumorigenesis [50]. Approximately 15% of patients with NF1 develop gliomas, mainly pilocytic astrocytomas, often occurring in the optic pathway [51]. The MAPK pathway plays a key role in the development of GBM by activating cell proliferation and transcription factor CREB, which regulates cyclin D-1 in GBM cells [52]. Furthermore, studies have shown that inhibiting the MAPK pathway inhibits the proliferation of GBM cells [53].

## 3. Role of PP2A in Neurodevelopment and Neurophysiology

The PP2A family of Ser/Thr phosphatases affects cellular pathways that are essential for a variety of neural processes, including neurodevelopment, stem-cell regeneration, neurotransmitter release, and postsynaptic responses. Over the past two decades, several knockout mouse models have highlighted the importance of PP2A in neural development. Liu et al. demonstrated that the cerebral knockout of the protein phosphatase 2ACα (PP2A-Cα) gene via the Cre-loxP targeting system resulted in cerebral cortical atrophy, marked neuronal shrinkage, synaptic plasticity impairments, and learning deficits [54]. Results suggested that PP2ACα downregulated the Hippo cascade in neural progenitor cells (NPCs) that is essential for normal neuronal growth and protein synthesis. Knockout of the PP2A-Cα gene also activated tumor suppressor p73 which in turn suppressed GLS2 activity and ultimately the glutamate–glutamine cycle, depriving NPCs of sufficient glutamine for protein synthesis. In a separate study, Yamashita et al. illustrated that PP2A-C and its regulatory protein α4 regulated hippocampal protein calmodulin kinase IIα (CAMKIIα), a kinase involved in memory, spatial learning, and long-term potentiation (LTP) [55,56]. In this study, a knockout mouse model inhibiting the expression of CNS α4 (NO-α4 KO) was prepared via conditional targeting. NO-α4 KO mice exhibited greater latency in both spatial and avoidance learning. Reducing PP2A activity increased hippocampal CAMKIIα activity in NO-α4 KO mice, providing evidence that PP2A/α4 suppresses CAMKIIα support of LTP and memory.

PP2A has been shown to regulate several other neurodevelopmental proteins, including GSK-3β, Dock6, tau, and collapsing response mediator protein II (CRMPII) [57,58,59,60]. PP2A also appears to be critically involved in axon morphogenesis. Miyamoto et al. showed that PP2A, in conjunction with protein kinase B (Akt), is responsible for Dock6-mediated axon extension and branching of dorsal root ganglion neurons [58]. A series of in vitro immunoassays of hippocampal rat neurons showed that PP2A complexes with Dock6 during initial axon development. Similarly, Zhu et al. identified the relationship between PP2A and CRMPII in axonal elongation [59]. PP2A was observed to dephosphorylate CRMPII at its Thr514 residue, activating the protein and enabling normal microtubule formation, actin reorganization, and axon protein shuttling in developing neurons.

The PP2A family of phosphatases has also been documented to modulate neurotransmitter release and postsynaptic responses [61]. Sim et al. first identified PP2A’s involvement in the neurosynaptic release of glutamate, aspartate, and GABA [61]. They found that the inhibition of PP2A via low molar okadaic acid in isolated rat synaptosomes increased the release of the aforementioned neurotransmitters by 34–89%. Later Beaulieu et al. identified PP2A as a regulator of Akt-mediated D2 class-receptor activity [62]. Using a functional and in vivo mouse model, PP2A was shown to contribute to an Akt/β-arrestin 2/PP2A scaffolding complex. This complex was determined to be a component of an alternative pathway for D2 receptor expression involved in dopamine-associated behaviors.

Among the proteins PP2A interacts with, perhaps the most extensively studied is tau. Tau is an axonal microtubule-associated protein shown to regulate microtubule dynamics, axonal transport, and signaling [63]. Tau has six isoforms and 37 Ser/Thr phosphorylation sites [64]. PP2A dephosphorylates the Ser/Thr tau sites, and accounts for 71% of all tau phosphatase activity in the brain, warranting its extensive study [65]. Sontag et al. first observed that PP2A/Bα binds directly to tau and dephosphorylates it [66]. A series of in vitro models using human NT2 neuronal precursor cells showed the direct interaction of PP2A/Bα and tau and that tau was hyperphosphorylated in the absence of PP2A/Bα. Schild et al. later demonstrated similar results in vivo: tau phosphorylation increased significantly when PP2A/Bα was downregulated [67].

Tau’s most recognized role in normal physiology is to regulate microtubular dynamics. Neurons affected by the aberrant hyperphosphorylation of tau cannot assemble microtubules, leading to axonal transport deficits and tau aggregations that manifest in a family of neurodegenerative diseases known as tauopathies [68]. As PP2A inhibition contributes to the hyperphosphorylation of tau, PP2A dysfunction can contribute to the pathogenesis and severity of several tauopathies, including Alzheimer’s Disease (AD), progressive supranuclear palsy (PSP), and Parkinsonism–dementia complex (PDC) [4,6,69,70]. PP2A prevents hyperphosphorylation of tau via the downregulation of several CNS Ser/Thr kinases involved in the pathogenesis of AD, including GSK-3β, JNK, and extracellular-regulated kinase (ERK) [71]. Sontag et al. observed dramatically decreased PP2A/Bα in brain areas affected by AD [6]. Furthermore, reduced levels of PP2A/Bα were inversely proportional to neurofibrillary tangle load in AD-affected neurons. Later, Park et al. demonstrated that PP2A contributed to the accumulation of hyperphosphorylated tau and the resulting neurodegeneration observed in tauopathies [69].

PP2A inactivation in cancer can be mediated by several processes, including somatic mutation, suppression of subunits, and upregulation of endogenous inhibitors [72,73,74,75,76,77]. As it is a master cellular regulator, PP2A activation has been identified as a strategy for tumor suppression in several oncological therapies [78].

Converse to PP2A’s homeostatic regulatory functions mentioned above, the isoforms PR72 and PR130 of the B” family of PP2A have been identified as tumor promoters, supporting prosurvival signaling and metastasis [48,79]. Recently, Tang et al. detailed the role of PP2A in regulating the Hippo–YAP signaling pathway in gastric cancer [80]. Dysregulation and subsequent loss of the tumor suppressor activity exhibited by Hippo has been indicated in a variety of cancers. Under normal conditions (Hippo turned on), Hippo kinases MST 1/2 phosphorylate LATS1 and LATS2 kinases, which then phosphorylate YAP and TAZ, causing the cytoplasmic sequestration of these proteins [81]. When the Hippo pathway is turned off, YAP and TAZ translocate to the nucleus and affect target gene transcription [82]. The dysregulation of this pathway has been implicated in cellular hyperproliferation and tumorigenesis [80]. Tang and colleagues demonstrated that PP2A-B subunit B’’’ (striatin family of proteins), specifically Striatin 3, recruits MST1/2, promoting its dephosphorylation and activating YAP [80]. Consequently, Striatin 3 upregulation has resulted in a poor prognosis in gastric cancer patients [80]. This highlights the role of PP2A as a potential oncogene, elucidating the importance of LB100 as a PP2A inhibitor. Janssens et al. identified that PR130 increased malignant cell migration and decreased cell-substratum adhesion when complexed with LIM protein lipoma-preferred partner (LPP) [79]. The loss of PR130 expression in HT1080 fibrosarcoma cells resulted in increased cellular adhesion to collagen while simultaneously decreasing cell migration in wound healing and transwell migration assays. In a separate study, Zwaenepoel et al. showed that PR130 prevented epidermal growth factor (EGF) receptor degradation, which resulted in sustained EGF-mediated signaling, driving the cellular proliferation, metastasis, and angiogenesis characteristics of many cancers [48].

In regard to a potential mechanism by which LB100 could be sensitive to DNA-damaging therapies, the loss of B55α function was observed in up to 40% of lung carcinomas. Kalev et al. observed that inhibition of B55α impaired homologous recombination (HR) DNA repair by inhibiting ATM and inducing G_1_-S phase cell cycle arrest [83]. Anticancer therapies were devised to radiosensitize cancer cells by inhibiting the B55α subunit of PP2A and preventing homologous recombination (HR) repair of radiation-induced DNA damage in tumor cells. Wei et al. confirmed that PP2A/Aα inhibition radiosensitized pancreatic cancer cells via the inhibition of HRR and hastened cell death following treatment [84]. In summary, PP2A serves as a potential target in cancer therapy given its positive regulatory roles in several signaling pathways that can bolster tumorigenesis and malignant disease characteristics.

## 4. LB100 as Therapy for Solid Nervous System Tumors

LB100 is derived from the synthetic anticancer compound and PP2A inhibitor norcantharidin, a homolog of the naturally occurring cantharidin used in traditional Chinese medicine [85,86]. It is a water-soluble small molecule that competitively inhibits PP2A by directly binding to PP2A-C and reducing its catalytic activity [87]. While improvements have been made to increase the specificity of protein phosphatase inhibitors, naturally occurring compounds, including cantharidin, only demonstrated relative specificity to PP2A and homologous serine/threonine phosphatases [88]. PP1, PP2B, PP4, PP5, PP6, and PP7 are among these homologs which share an extensive similarity in their catalytic centers to PP2A-C [88,89]. This highlights the importance of understanding the bioactivity of LB100 as an inhibitor of abundant protein phosphatases other than PP2A. Further studies are likely warranted to elucidate the inhibitory potential of LB100 among phosphatases that have tumor suppressor or oncogenic functions.

For example, PP5 has been shown to be overexpressed in a variety of mammalian cancers, including glioma and osteosarcoma [90,91,92]. Therefore, inhibition of the catalytic activity of PP5 could have therapeutic potential. Interestingly, LB100 has recently emerged as a potential inhibitor of PP5. In addition to binding to PP2A-C, LB100 can bind to and inhibit PP5C [93]. PP2A-C and PP5C share a common catalytic mechanism, evidenced by the significant structural homology of these enzymes’ catalytic pockets [93]. Initial preclinical and clinical studies have shown that LB100 is efficacious in targeting tumors of the nervous system, entering the tumor through the permeable blood–tumor barrier. Another direct inhibitor of PP2A, LB102, is a lipid-soluble homolog of LB100 that has antitumor efficacy demonstrated by the chemosensitizing of GBM cells to DNA-alkylating agents [10,94]. LB100 and LB102 inhibit PP2A similarly, but LB102 has greater lipid solubility, affording it greater blood–brain barrier penetrance and higher drug levels in brain tissue surrounding the main tumor mass [10].

### 4.1. Glioblastoma

Glioblastoma is the most common primary malignant brain tumor and is highly aggressive. Despite research revealing its genetics, epigenetics, and molecular pathogenesis, therapeutic advances have been limited and its prognosis remains dismal. On average, patients survive 12–15 months after GBM diagnosis. GBM survival is prolonged somewhat by surgery, radio-, and chemotherapy, but these measures cannot stop the disease progression, which is thought to arise from the ability of subsets of tumor stem cells to self-renew and resist chemotherapeutic agents. A better understanding of the mechanisms by which tumor stem cells evade chemo- and radiotherapy may elucidate molecular pathways that can be targeted to prevent tumor stem cell chemo- and radioresistance. One such investigation was conducted by Lu et al. who studied the effect of LB100 on the nuclear receptor corepressor (N-CoR) pathway (Table 2) [95]. This pathway has a putative role in preserving the immortal nature of undifferentiated tumor stem cells and is overexpressed in GBM [96]. Inhibition of PP2A with LB100 led to decreased expression of N-CoR, increased levels of phosphorylated Akt kinase, and decreased tumor cell proliferation in U87 and U251 malignant glioma cell lines [95]. In vivo experiments in mice with U87 glioma xenografts demonstrated that systemic treatment with LB100 reduced mean tumor volume by 73% (*p* < 0.001) compared to controls [95]. The same group followed up these studies by investigating the effects of PP2A inhibition with LB100 on U251 GBM cells in vivo and in vitro following radiation treatment [97]. They reported that LB100 administration sensitized GBM cells to the cytotoxic effects of radiation in vitro and significantly delayed tumor growth in vivo after radiation (*p* < 0.001) [97]. Recently, another study reported combining LB100 and chimeric antigen receptor (CAR)-engineered T cells to treat GBM (Table 2) [98]. Cui et al. initially tested the combination of CAR-engineered T cells targeting carbonic anhydrase IX (CAIX), a protein involved in hypoxic signaling, and LB100 in GBM cell lines [98]. Combination therapy in vitro demonstrated a synergistic effect of LB100 and CAR-T cells and a significant increase in cytotoxic markers. Later, in vivo experiments in the U251-Luc glioma mouse model showed that combination therapy significantly increased tumor-infiltrating lymphocytes (TILs) in harvested tumors (*p* < 0.05) and overall survival. This suggested that LB100 was effective in reducing the tumor burden in GBM and could also synergize with immunotherapeutics. Maggio et al. investigated therapy for GBM using LB100 combined with immune checkpoint inhibitor PD1 (Table 2) [99]. They implanted mice with glioma cell lines, administered the combination therapy, and assessed overall survival and tumoral penetration of TILs. LB100 and PD1 blockade combination therapy significantly increased survival compared to monotherapy alone (*p* < 0.05). Furthermore, tumors regressed completely in 25% of mice in the combination treatment group, whereas this was not the case for any of the mice in the monotherapy treatment groups or vehicle-only controls. Flow cytometry performed on the tumor-harvested TILs demonstrated a significant increase in CD8+ T-cells in the combination therapy group compared to controls, LB100 monotherapy cohort, and PD1 blockade alone cohort (*p* < 0.0005, *p* < 0.005, and *p* < 0.05, respectively) [99]. Most recently, our group studied the effects of combining LB100 with the knockdown of protein arginine methyltransferase 5 (PRMT5). For these experiments, patient-derived primary glioblastoma neurospheres (GBMNS) were initially transfected with PRMT5 siRNA (Table 2) [100]. LB100 administration in conjunction with PRMT5 knockdown was then studied in the in vitro and in vivo intracranial mice xenografts models. We found that LB100 significantly reduced the viability of PRMT5-depleted GBMNS by inducing necroptosis. In vivo studies showed that combination therapy significantly decreased tumor volume and prolonged the survival of mice compared to the PRMT5 knockdown and control groups. LB100 alone did not increase the survival of GBMNS implanted mice. Overall, LB100 may increase the therapeutic effects of other available therapeutic agents against GBM.

### 4.2. Pheochromocytoma

While LB100 has been better studied for GBM therapy, it has also been explored as a chemotherapeutic adjunct in other central and peripheral nervous system tumors. One such example is the treatment of the highly drug-resistant neuroendocrine tumor, pheochromocytoma. Pheochromocytomas are slow growing, and their low growth fraction contributes to their high resistance to chemo- and radiotherapy [101]. While most pheochromocytomas and paragangliomas are benign, reports estimate that 11–31% of patients present with or develop metastatic disease [102]. Martiniova et al. explored whether combining LB100 with the monofunctioning alkylating prodrug Temozolomide (TMZ) could improve TMZ’s effectiveness, applying this combination therapy on a pheochromocytoma cell line and in an in vivo mouse model (Table 2) [101]. TMZ alkylates single stranded DNA at specific locations to produce O6-methylguanine nucleotides, which results in thymine insertion instead of correctly inserting cytosine. This results in the formation of single and double stranded DNA breaks leading to G2/M cell cycle arrest and, subsequently, tumor cell apoptosis [103].

The LB100 and TMZ combination therapy studied in vitro modestly reduced pheochromocytoma cell proliferation. In the in vivo model, mice developed metastatic tumors. Combination therapy resulted in complete tumor remission in 40% of these mice and significantly delayed hepatic tumor growth in the remainder [101]. Histological sectioning of treated liver pheochromocytoma revealed extensive tumor necrosis. The in vivo tumor remissions and delay in hepatic tumor growth in this initial study of the effect of LB100 combination therapy on pheochromocytomas suggest that LB100 deserves further investigation as an adjunct to TMZ in treating metastatic pheochromocytoma.

### 4.3. Medulloblastoma

Medulloblastoma (MB) is the most common primary malignant brain tumor in the pediatric population, accounting for nearly 20% of all CNS tumors in children [104,105]. The standard treatment regimen for these tumors involves maximal gross total resection followed by radiotherapy and chemotherapy [106]. Some molecular and histological variations of MBs have favorable prognoses, but patients with group 3 and/or recurrent tumors have poor overall survival [107]. Treatment options for patients with recurrent tumors are limited because these neoplasms are often resistant to chemotherapy and radiation, and novel therapeutic approaches are sorely needed. Ho et al. studied the effect of LB100 and the alkylating platinum analog cisplatin on MB cells in vitro and in vivo in an intracranial xenograft mouse model (Table 2) [87]. Cisplatin induces DNA crosslinks which prevents repair, blocks cell division, and causes apoptotic cell death [108]. In vitro, LB100 monotherapy increased apoptosis in a dose-dependent manner in two MB cell lines and also induced G2/M cell cycle arrest. LB100 monotherapy significantly decreased MB cell migration compared to cisplatin monotherapy and untreated control cells (*p* < 0.05). Combination therapy with LB100 and cisplatin significantly increased the number of apoptotic tumor cells compared to controls (*p* < 0.05). In vivo experiments confirmed the therapeutic efficacy of the LB100 and cisplatin combination observed in MB cell lines. Combination therapy reduced tumor growth, measured 64 days after implantation, significantly more than cisplatin therapy alone (*p* < 0.05). The potent in vivo antineoplastic activity of the LB100 and cisplatin combination warrants further investigation.

### 4.4. Diffuse Intrinsic Pontine Glioma

Diffuse intrinsic pontine glioma (DIPG) is the most common pediatric brainstem tumor and has a uniformly fatal prognosis [109]. Radiation therapy is the only treatment with some efficacy against DIPG, extending average survival only by a few months. The discovery and development of novel therapeutics for this devastating childhood cancer is paramount. To identify DIPG susceptibilities, Schramm et al. used a pooled short hairpin RNA library, next-generation sequencing, and a large-scale gene knockdown approach (Table 2) [110]. These investigators identified fibroblast growth factor receptor signaling and PP2A as the top depleted hits and important potential targets for inhibition [110]. They found in two DIPG cell lines that LB100 treatment for 2.5 h induced phosphorylation of Akt and cell cycle regulator PLK1 and that LB100 treatment for 50 h induced apoptosis in a dose-dependent manner. The involvement of PP2A in the regulation of apoptotic and proliferative signaling pathways in DIPG tumor samples suggests that PP2A targeting should be explored further as a primary and adjunctive therapeutic agent for DIPG.

### 4.5. Neuroblastoma

Neuroblastoma has variable clinical behavior, in which some tumors spontaneously regress while others progress even with aggressive therapy regimens. Despite advances in multimodal therapy, achieving therapeutic success in neuroblastoma is often a challenge. Surgery and chemotherapy are usually used initially and may be curative. The prognosis for children with high-risk neuroblastoma variants is poor, necessitating innovative treatment options. In patients unresponsive to standard measures, novel agents may be employed, most of which target the *MYCN* oncogene and proangiogenetic factors [111]. Lu et al. studied the antitumor effects of PP2A inhibitor LB102 (lipid-soluble homolog of LB100) in combination with temozolomide (TMZ) in neuroblastoma (NB) and GBM mouse xenograft models (Table 2) [94]. The investigators found that PP2A inhibition increased the antitumor activity of TMZ and completely suppressed tumor growth in mice implanted with NB xenografts. This study demonstrated the chemosensitizing properties of LB100 when given with TMZ, an alkylating chemotherapeutic agent.

## 5. Biological Insights and Future Directions

LB100 use as an adjuvant chemotherapeutic agent has advanced from preclinical in vitro and in vivo experiments to clinical trials. One phase I clinical trial of LB100 in the treatment of solid tumors has been completed (ClinicalTrials.gov Identifier: NCT01837667) and achieved promising results [112]. This study, conducted by Chung et al., was an open-label, dose-escalation phase I trial where 29 study participants with progressive solid tumors received intravenous doses of LB100 daily for 3 days, administered in 21-day cycles [112]. Ten of the twenty patients who were available to respond achieved stable disease for four or more cycles. Two patients had dose-limiting toxicity, and one patient who initially stopped treatment due to an acute infection was later reenrolled. The objective of this initial trial was to determine the maximum tolerated dose of LB100 while also evaluating its safety and activity. Interestingly, LB100 was administered to trial subjects as a monotherapy. Currently, evidence has indicated that LB100 exerts its antitumor effects primarily through chemo- and radiosensitization [10]. This idea was supported by the aforementioned study conducted by Lu et al. who showed the chemosensitizing effects of an LB100 analog on neuroblastoma and GBM cells [94]. Treatment with LB-1.2 was associated with increased phosphorylation of MDM2 and decreased phosphorylated p53.

MDM2 functions to inhibit the tumor suppressor activity of p53 [113]. However, reports have revealed that MDM2 may, in fact, positively regulate p53 under certain conditions, such as after ATM-dependent MDM2 phosphorylation which enhances p53 translation. This is particularly relevant to GBM tumor biology given that the p53-ARF-MDM2 pathway is reported to be deregulated in 84% of patients with GBM [113]. The rescue of this pathway via LB100-induced phosphorylation of MDM2, with subsequent upregulation of tumor suppressor p53, highlights a potential antitumor mechanism that likely warrants further study and clinical translation.

Since the initial clinical trial by Chung et al., another trial evaluating the efficacy of LB100 in the treatment of recurrent GBM is ongoing and still recruiting patients (ClinicalTrials.gov Identifier: NCT03027388). This is a two-stage, phase II, open-label trial which aims to delineate the pharmacodynamics and pharmacokinetics of LB100 monotherapy in patients with recurrent high-grade gliomas. Furthermore, this study will serve to determine whether LB100 can cross the blood–brain barrier (BBB) in humans and at what plasma concentrations LB100 can penetrate the BBB at an effective therapeutic dose. With the proper characterization of the BBB penetration profile of LB100, treatment can be clinically optimized for its administration with additional chemotherapeutic agents. While progress has been made in evaluating its anticancer properties in early phase clinical trials, a significant research gap exists concerning the possible role that LB100 could play in improving the treatment of solid nervous system tumors.

## 6. Conclusions

GBM and most other CNS gliomas defy cure because of their unique tumor biology and invasion into anatomical regions of eloquent neurological function. Better treatment of these tumors is needed and will result from advancements in neuro-oncologic research identifying tumor susceptibilities that may be targeted with new therapeutic agents, such as LB100 and other PP2A inhibitors. Further investigations will elucidate the efficacy of this drug in reducing the disease burden of peripheral and central nervous system neoplasms.

## Figures and Tables

**Table 1:** List of known PP2A subunits including additional associated names. Nervous system expression represents the highest level of PP2A subunit gene expression in a central nervous system structure. Genetic information was obtained from the UniProt protein sequence database (https://www.uniprot.org/ (accessed on 3 June 2021)).

Subunit Family	Protein Isoform	Other Associated Name(s)	Nervous System Tissue Expression
A	Aα	PR65α	Anterior cingulate cortex
	Aβ	PR65β	Corpus callosum
B”’/Striatin	B”’	Striatin	Corpus callosum
B/PR55	Bα	B55α/PR55α	Corpus callosum
	Bβ1	B55β1/PR55β1	Corpus callosum
	Bβ2	B55β2/PR55β2	Corpus callosum
	Bγ	B55γ/PR55γ	Caudate nucleus
	Bδ	B55δ/PR55δ	Dorsal/ventral thalamus
B’/PR61	Bα	B56α/PR61α	Corpus callosum
	Bβ	B56β/PR61β	Right cerebellar hemisphere
	B’γ1	B56γ1/PR61γ1	Caudate nucleus
	B’γ2	B56γ2/PR61γ2	Caudate nucleus
	B’γ3	B56γ3/PR61γ3	Caudate nucleus
	B’δ	B56δ1/PR61δ	Dorsal/ventral thalamus
	B’ε	B56ε/PR61ε	Forebrain
B”/Pr72	B”α	PR130	Forebrain
	B”α	PR72	Forebrain
	B”β	PR70	Hypothalamus
	B”γ	G5PR	C1 segment of cervical spinal cord
C	Cα	PP2Acα	Frontal cortex
	Cβ	PP2Acβ	Dorsal/ventral thalamus

**Table 2:** Overview of reviewed studies involving the use of LB100 as monotherapy or combination therapy against tumors of the nervous system. CAIX = Carbonic Anhydrase IX; CAR = Chimeric Antigen Receptor; DIPG = Diffuse Intrinsic Pontine Glioma; FGFR = Fibroblast Growth Factor Receptor; GBM = Glioblastoma; NB = Neuroblastoma; NCoR = Nuclear Receptor Corepressor 1; PHEO = Pheochromocytoma; POD = Postoperative Day; PRMT5 = Protein arginine methyltransferase 5.

Investigators (Year)	Tumor Type	Treatment Method	Outcome
Lu et al. [95] (2010)	Glioblastoma	LB100 only	LB100 inhibited PP2A and caused dose-dependent antiproliferative activity in two GBM cell lines. LB100 treatment resulted in a significant reduction in tumor volume compared to controls (*p* < 0.001) in vivo. In vivo experiments also resulted in decreased nuclear N-CoR expression.
Gordon et al. [97] (2015)	Glioblastoma	LB100 and radiation therapy	LB100 resulted in radiation dose enhancement and increased mitotic catastrophe. Combination therapy significantly enhanced tumor growth delay while decreasing p53 in vivo. Combination therapy also increased the overall survival of mouse xenografts.
Cui et al. [98] (2020)	Glioblastoma	LB100 and CAR-T cells	Anti-CAIX CAR-T cell and LB100 combination therapy resulted in significant cytotoxicity against GBM tumor cells and increased cytokine production compared to control T-cell treatment in vitro. Combination therapy significantly increased tumor regression compared to monotherapy in vivo (*p* < 0.05) and significantly prolonged survival (*p* < 0.001).
Maggio et al. [99] (2020)	Glioblastoma	LB100 and PD-1 inhibition	Combination therapy significantly improved survival compared to monotherapy (*p* < 0.005) and controls (*p* < 0.001). Complete tumor regression was seen in 25% of combination-treated mice but no other subgroups.
Otani et al. [100] (2021)	Glioblastoma	LB100 and PRMT5 knockdown	LB100 administration significantly reduced viability in PRMT5-depleted GBMNS compared to PRMT5-intact GBMNS. PRMT5 knockdown and LB100 combination therapy increased the expression of phospho-MLKL. Combination therapy significantly decreased tumor size and prolonged survival in in vivo mouse xenografts.
Lu et al. [94] (2009)	Neuroblastoma and GBM	LB102 and TMZ	LB102 treatment in U87MG GBM cells resulted in morphological features of mitotic catastrophe. LB102 caused complete regression of GBM xenografts with no recurrence in 50% of animals and inhibited the growth of NB xenografts.
Martiniova et al. [101] (2011)	Pheochromocytoma	LB100 and TMZ	Combination therapy resulted in significantly greater tumor cell inhibition in vitro compared to monotherapy. PHEO mouse xenografts treated with combination therapy had significantly prolonged survival compared to monotherapy (*p* < 0.0001). Combination therapy significantly delayed the appearance of hepatic tumors compared to monotherapy alone (*p* < 0.0001).
Ho et al. [87] (2016)	Medulloblastoma	LB100 and cisplatin	LB100 alone had a potent antitumor effect of multiple medulloblastoma cell lines. Combination therapy enhanced cisplatin cytotoxicity and significantly decreased medulloblastoma cell viability as compared to controls (*p* < 0.005). Combination therapy significantly reduced tumor burden on POD64 compared to cisplatin treatment alone (*p* < 0.05) in vivo.
Schramm et al. [110] (2019)	DIPG	LB100 only	Investigators used a large-scale gene knockdown approach using shRNA and DNA sequencing to identify susceptibilities of DIPG tumor cells. Screening resulted in FGFR and PP2A deemed as candidate targets. LB100 therapy induced apoptosis in two DIPG cell lines in a dose-dependent manner and increased pAkt expression in vitro.

## Data Availability

Not applicable.
